# Ocean acidification changes the male fitness landscape

**DOI:** 10.1038/srep31250

**Published:** 2016-08-17

**Authors:** Anna L. Campbell, Don R. Levitan, David J. Hosken, Ceri Lewis

**Affiliations:** 1 College of Life and Environmental Sciences, University of Exeter, Geoffrey Pope Building, Stocker Road, Exeter, EX4 4QD, UK; 2 Department of Biological Science, Florida State University, King Life Sciences Building, 319 Stadium Drive, Tallahassee, FL, 32306-1100, USA; 3 College of Life and Environmental Sciences, University of Exeter, Cornwall CampusTreliever Road, Penryn, Cornwall, TR10 9FE, UK

## Abstract

Sperm competition is extremely common in many ecologically important marine taxa. Ocean acidification (OA) is driving rapid changes to the marine environments in which freely spawned sperm operate, yet the consequences of OA on sperm performance are poorly understood in the context of sperm competition. Here, we investigated the impacts of OA (+1000 μatm *p*CO_2_) on sperm competitiveness for the sea urchin *Paracentrotus lividus*. Males with faster sperm had greater competitive fertilisation success in both seawater conditions. Similarly, males with more motile sperm had greater sperm competitiveness, but only under current *p*CO_2_ levels. Under OA the strength of this association was significantly reduced and there were male sperm performance rank changes under OA, such that the best males in current conditions are not necessarily best under OA. Therefore OA will likely change the male fitness landscape, providing a mechanism by which environmental change alters the genetic landscape of marine species.

Seawater conditions are currently changing at a rate faster than at any other time for the past 300 million years^1^, as rising atmospheric carbon dioxide (CO_2_) levels modify seawater chemistry and decrease ocean pH^2^ (termed OA). The unprecedented rate of change is likely to place significant novel selection on marine taxa. Negative impacts of OA on growth, reproduction or survival have been observed in a broad range of species^3^,^4^ and OA is now widely considered a major threat to marine taxa worldwide^5^. The majority of marine species release sperm and eggs directly into the water column for external fertilisation, including several large taxa with key roles in ecosystem functioning^6^,^7^. The sperm of external fertilisers may be particularly vulnerable to OA due to the limited buffering capacity of internal-sperm pH against changes to seawater pH^8^, together with the large number of pH-dependent steps taking place inside the sperm cell that are critical to fertilisation^9^. Consistent with this, the majority of studies of external fertilisers have identified reductions in sperm swimming speeds and/or the proportion of motile sperm in at least one of their OA treatments^10^,^11^,^12^,^13^. However, some studies have found no effect^14^ and even motility enhancement under OA^15^, which could indicate species-specific sperm sensitivity to OA or simply methodological differences between studies.

Sperm are under intense selection to achieve fertilisation, as in most species only a tiny fraction of the millions of sperm in an ejaculate succeed in fertilising an egg^16^. But being fertilisation competent is often not enough, because in the vast majority of sexually reproducing species sperm from rival males compete for fertilisations^17^. Sperm competition is practically ubiquitous across the animal kingdom^16^ and results in strong selection on males to produce high quality ejaculates^17^. Sperm number, length, swimming velocity and viability all influence sperm competitiveness in internal fertilisers and pair spawning fish^18^. These characteristics should also influence sperm competition outcomes in marine external fertilisers. However, this area has received little research attention and experimental evidence to link sperm phenotypes to competitive fertilisation success is scarce in this ecologically important group. Under non-competitive scenarios, sperm concentration^19^, age (time in seawater post-spawning)^20^, swimming velocity and longevity^21^ all influence male reproductive success. However, male external fertilisers rarely gain sole access to a batch of eggs in the sea as a consequence of life-history strategies which include spawning aggregations and the synchronous release of gametes^22^. Despite this, our understanding of the male traits influencing sperm competition outcomes remains poor in external fertilisers. Changes to seawater chemistry resulting from OA will fundamentally alter the fertilisation environments in which marine sperm operate. Therefore it is becoming increasingly important to identify the factors influencing competitive fertilisation success in marine external fertilisers, and to identify any changes that could occur as a result of OA. Selection at the gamete stage can have far-reaching consequences for populations, carrying over to subsequent life stages^12^ and to date we have little information on sperm performance in future oceans. The importance of including environmental effects on sperm ecology in order to understand species evolutionary responses was recently highlighted by Reinhardt, *et al*.^23^. Their review presented overwhelming evidence that the environment can influence sperm, and highlighted pH as one of the key environmental factors that can influence phenotypic sperm function across species. Here, we address this key area of research by exploring the repeatability of the outcome of sperm competition across two environments.

We conducted a series of paired competitive fertilisation trials under current ocean conditions (pH 8.18, 462 μatm *p*CO_2_) and simulated future OA conditions (pH 7.71, 1468 μatm *p*CO_2_) in the sea urchin *Paracentrotus lividus*. Male ejaculate characteristics were evaluated in each seawater treatment and, based upon average sperm swimming speeds (curvilinear velocity: VCL) in current seawater conditions, males were split into a ‘fast’ and a ‘slow’ group. Each ‘fast’ male was randomly paired with a ‘slow’ male generating n = 11 pairs (average speed difference ±95% confidence interval = 42.78 μm s^−1^ ± 12.51). Each pair competed to fertilise a batch of eggs (n = 10,000) in both current and OA seawater treatments at a total sperm concentration of 1 × 10^5^ sperm ml^−1^ (and an equal number from each male). The resulting larvae were genotyped using microsatellites to assign paternity.

## Results and Discussion

**Figure 1:**
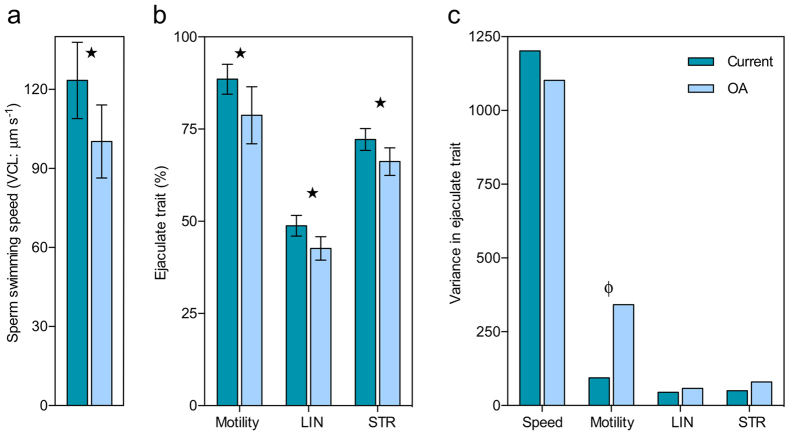
Ejaculate traits in current and OA seawater conditions. (**a**) Male sea urchin (n = 22) ejaculate performance in seawater treatments (group means ± 95% confidence intervals) and (**b**) the variance observed in each ejaculate trait. *Indicates the significant reduction in all ejaculate traits under OA conditions and ϕindicates the significant increase in variance in sperm motility under OA conditions (*p* ≤ 0.05). LIN = average sperm path linearity and STR = average sperm path straightness.

Marine sperm are generally stored immotile in the gonad. Upon release into seawater, the change in extrinsic environmental conditions triggers a chain of events leading to activation and the initiation of swimming. Intracellular pH plays a crucial role in the activation of marine invertebrate sperm swimming^24^ and determines the activity of enzymes which produce ATP to power swimming and drive flagella beating which propels a sperm forward^9^. pH can differentially affect freshwater sperm^25^ but the natural ranges of pH for freshwater are much wider than relevant for seawater or OA. Internal sperm pH is presumed to be vulnerable to changes in seawater pH as sperm are single cells and have a greatly reduced cytoplasm which is thought to limit their pH buffering capacity. Consistent with this we found that our experimental OA conditions significantly changed sperm performance. Average swimming speeds were reduced by 18.8% (Fig. 1a. Paired t-test: t = 3.692, df = 21, *p* = 0.001), and 9.8% fewer sperm in an ejaculate were classed as motile (Fig. 1b. Wilcoxon signed-rank test: V = 3.088, df = 21, *p* = 0.015). Both ejaculate traits are important in models of population fertilisation ecology in external fertilisers^26^. The reductions we observed could potentially reduce population level fertilisation rates with negative implications for population size and/or persistence.

Marine invertebrate sperm swim in circular or helical paths^27^. Physical models have revealed that this enables them to sense gradients in chemical cues released by unfertilised eggs most effectively^28^. Following an initial search phase, if a sperm detects such a gradient, it then navigates towards the source in a phenomenon known as chemotaxis^29^. We assessed sperm swimming behaviour using computer assisted sperm analysis (CASA) and found significant reductions under OA in two CASA derived parameters which measure the linearity of sperm swimming paths; average path linearity (LIN: Fig. 1b. Paired t-test: t = 3.037, df = 21, *p* = 0.006) and average path straightness (STR: Fig. 1b. Paired t-test: t = 3.320, df = 21, *p* = 0.003). Whilst the exact repercussions of these reductions under OA are currently unknown, any change has the potential to influence fertilisation rates through alterations in sperm searching efficiency. Fitzpatrick, *et al*.^30^ demonstrated the selective importance of curved sperm swimming paths for maximising fertilisation success, providing further evidence that this character influences male fitness. Although variance remained unchanged across seawater treatments in most ejaculate traits, there was a significant increase in variance in the percentage of motile sperm in a male’s ejaculate under OA (Fig. 1c. Levene’s test: F = 13.264, df = 41, *p* = 0.001. Supplementary Table S1). So, in addition to an overall reduction in percentage sperm motility across males, differences between males were also amplified under OA conditions which could have implications for male competitiveness.

**Figure 2:**
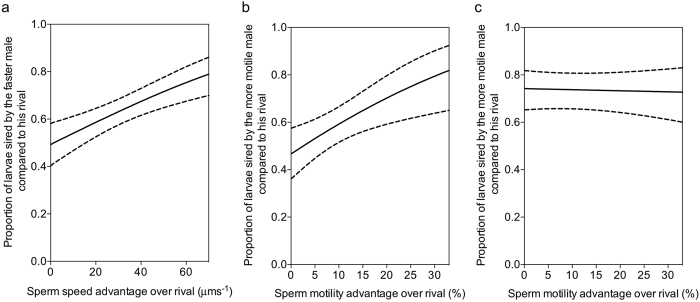
The GLMM modelled influence of relative male ejaculate traits on competitive fertilisation success. The modelled relationship between (**a**) average sperm swimming speed (VCL) and secondly the percentage of motile sperm in a male’s ejaculate under current seawater conditions (**b**), and under future OA conditions (**c**), and the proportion of larvae sired by the focal male in paired competitive fertilisation trials (n = 11 pairs) in the sea urchin *Paracentrotus lividus*. Predictions ± 95% confidence intervals were calculated using the ‘best fitting’ GLMM with all other model parameters kept at their observed median values.

We selected the generalized linear mixed-effects model (GLMM) which was most supported by the observed paternity share dataset (see Methods and Supplementary Tables S2–S5). The ‘best fitting’ model contained the following fixed effects; relative average sperm swimming speed (VCL) and an interaction between the seawater conditions and relative percentage sperm motility. This model was slightly underdispersed (model dispersion: 0.541). Results from our competitive fertilisation trials revealed a positive relationship between sperm swimming speed and paternity shares (Fig. 2a. GLMM: *p* = 0.000. Table 1). Males with a speed advantage over their rival fertilised a greater proportion of the batch of eggs, and their relative reproductive success increased with larger speed advantages. Similar results have been reported in pair spawning fish^31^,^32^ and internal fertilisers^33^,^34^ but see ref. 35 providing tentative evidence that sperm swimming speed might provide a selective advantage under sperm competition across reproductive modes. This positive relationship between speed and competitive fertilisation success held across seawater conditions. This provides previously missing empirical support for a long-held paradigm; that faster swimming speeds enhance sperm competitiveness in external fertilisers. Models containing the average swimming speed of the fastest 1, 5 or 10% of motile sperm were not most supported by the observed paternity shares. Hence, the results from our trials do not provide support for an association between the faster sub-populations of sperm within a male’s ejaculate and competitive fertilisation success.

The competitive fertilisation trials also revealed that paternity share was influenced by the percentage of motile sperm in a male’s ejaculate (Fig. 2b). In current ocean conditions this meant ejaculates with more motile sperm secured greater paternity, a result that seems intuitive and aligns with the body of literature on sperm concentration and fertilisation success collected under non-competitive scenarios^19^,^36^. Interestingly, we identified a significant interaction between seawater conditions and the influence of percentage sperm motility on paternity (GLMM: *p* = 0.010. Table 1). Under OA conditions, the relationship between the percentage of motile sperm in an ejaculate and a male’s competitive fertilisation success significantly weakened (Fig. 2c). The modelled data revealed that whilst males with more motile sperm than their rival still fertilised the majority of a batch of eggs (>50%) in OA conditions, the positive relationship between this ejaculate feature and paternity is lost. The consequences of this are far from clear, but changing the relationship between an ejaculate trait and male reproductive success under OA means OA is altering selection on males and their ejaculates.

**Figure 3:**
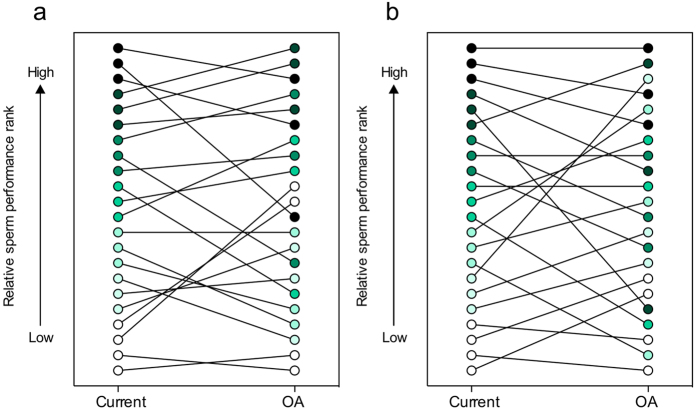
Male sperm performance ranks in current and OA seawater conditions. Male sea urchins (n = 22) ranked by (**a**) the percentage of motile sperm in an ejaculate and (**b**) average sperm swimming speed (VCL). Points are coloured by rank in ambient seawater (darker colours represent higher ranks in ambient conditions i.e. faster speed or greater motility) and male ranks in the two seawater treatments are connected by lines.

We compared male performance ranks across seawater treatments to investigate whether relative ejaculate performance in current seawater conditions correlated with performance under OA. We found a significant correlation between male ranks by the percentage of motile sperm in their ejaculate between current and OA seawater conditions (Fig. 3a. Spearman’s rank correlation: r_s_ = 0.730, df = 20, *p* = 0.000). Despite this correlation, there were many rank order changes illustrated by the crossing over of lines in Fig. 3a. When males were ranked by the average speed of the motile sperm in their ejaculate, there was a similar association (Fig. 3b. Spearman’s rank correlation: r_s_ = 0.614, df = 20, *p* = 0.003), but once again there were many rank order changes. This, together with the results of the competitive fertilisation trials, suggests that the identity of high fitness males could change as OA progresses. The positive relationship between the percentage of motile sperm in a male’s ejaculate and competitive fertilisation success was lost under OA conditions. So, despite the correlation in male ranks for this ejaculate trait across seawater conditions, males with the greatest percentage of motile sperm might not secure the highest relative reproductive success under OA. The positive relationship between sperm swimming speed and paternity shares won in competition held across seawater conditions, but despite this association, there were substantial rank order switches which could generate changes in the identity of males securing reproductive success in future oceans.

A significant proportion of the paternity variation we observed could not be explained by a model composed of relative male ejaculate characteristics and our treatment seawater conditions. Both the fixed and random terms included in the ‘best fitting’ GLMM only explained 11.04% of variation in the observed paternity shares. Therefore, there must be other parameters which contributed to sperm competition outcomes under the conditions of our fertilisation trials, which were not measured in our study. One accepted source of variation in sea urchin fertilisation rates is differences in gamete compatibility^37^. Gamete recognition proteins help sperm and eggs to identify one another and fuse^38^. Eggs can show strong affinities to sperm with particular recognition protein genotypes, generating differences in male fertilisation rates^39^, but we did not assess this here. In addition to roles in sperm activation and determining the activity of enzymes involved in sperm swimming, intracellular sperm pH is known to be involved in other processes essential for fertilisation in marine invertebrates. These include sperm response to egg chemical cues, which either act to enhance sperm movement or aid sperm navigation towards an egg^40^, and the acrosome reaction^41^. Thus there is clear potential for additional OA impacts beyond those we measured here.

Seawater *p*CO_2_ is more variable in coastal waters than open oceans^42^, and is often elevated in benthic habitats compared to surface waters where values of up to 2500 μatm have been recorded^43^. High resolution data on current seawater pH and *p*CO_2_ values for coastal benthic environments is limited and rarely linked to the location and timing of marine invertebrate spawning events, as it can be challenging to observe these often unpredictable and rare events. There is no current data on the seawater carbonate chemistry during a *P. lividus* population spawning event to provide us with details of the environmental conditions that their sperm currently compete within and inform our OA treatment conditions. Given this, we selected an OA treatment level for our study based upon the lower range of pH values projected under the Representative Concentration Pathway (RCP) 8.5 scenario for the year 2100^44^ in line with other studies on coastal benthic species. The conditions for sperm competition in complex natural fertilisation environments will be far more variable than in our simplified laboratory setup^45^,^46^, but our results provide a valuable first insight into the reproductive consequences of OA for external fertilisers under conditions of sperm competition. Populations of *P. lividus* may spawn into seawater conditions approximating our OA treatment within a relatively short timeframe (~100 years), which given the generation time of this species could limit the potential for evolutionary responses.

We have provided novel evidence that OA influences competitive interactions between males during fertilisation. We found that OA conditions reduced fundamental sperm performance parameters, caused some switching of male ranks by relative sperm performance and changed the influence of an ejaculate characteristic on sperm competitiveness. These changes are likely to be the tip of the iceberg with additional cascading effects yet to be identified. Importantly, the identity of competitive males and the male trait combinations important for fitness are likely to change with OA, and hence we might expect a shift in the fitness landscape for males under future ocean conditions.

## Methods

### Assessment of ejaculate characteristics

Adult urchins (Dunmannus Seafood Ltd., Ireland) were induced to spawn via KCl injection^21^ with sperm collected dry prior to use. Sperm was activated in each seawater condition and incubated for 10 minutes at 14 ± 0.1 °C (see Supplementary Methods for details of seawater *p*CO_2_ manipulation and Supplementary Table S6). Ejaculate characteristics were then measured within each seawater treatment using Computer Assisted Sperm Analysis (CASA) and the methodology described in Campbell, *et al*.^47^ (see also Supplementary Methods). Immotile sperm were defined as sperm swimming below threshold values of 10 μm s^−1^ curvilinear velocity (VCL) and 3.2 μm s^−1^ straight line velocity (VSL). The average sperm swimming speed (VCL) was then calculated for all motile sperm within a sample. Averages of two additional CASA parameters were calculated for all motile sperm within a sample; sperm path linearity (LIN) and sperm path straightness (STR). LIN and STR are measures of the linearity of sperm swimming paths and are calculated by the CASA software (see Supplementary Methods). Higher values of either LIN or STR indicate more linear sperm swimming paths i.e. a sperm is progressively motile.

The sperm swimming data was visually checked for normality, which was further confirmed via Shapiro-Wilk normality tests. The influence of OA on ejaculate traits was assessed using paired t-tests and variance in traits compared across seawater treatments using Levene’s median tests. As the percentage sperm motility data significantly deviated from a normal distribution, the influence of OA on this ejaculate trait was assessed via the non-parametric equivalent of the paired t-test: the Wilcoxon signed-rank test. Males were ranked by ascending sperm performance in current and OA seawater conditions and the strength of correlation between male ranks in the two seawater treatments was examined using the Spearman’s rank correlation coefficient.

### Competitive fertilisations and larval paternity assignment

Each pair of males competed to fertilise the eggs (n = 10,000) of a single female in the two seawater treatments. Eggs were obtained from a total of 6 females. Competitive fertilisation trials were repeated with the eggs of additional females (n = 3) to reduce the influence of fertilisation biases generated by differences in gamete compatibility. Sperm from each pair was activated in seawater of the appropriate treatment, mixed, and immediately added to the eggs and treatment seawater at a final sperm concentration pf 1 × 10^5^ sperm ml^−1^. This sperm concentration was selected to avoid conditions of both sperm limitation and polyspermy based upon data collected for another sea urchin species *Strongylocentrotus franciscanus*^48^,^49^. Fertilisation beakers were incubated overnight at 14 ± 0.1 °C. The resulting larvae were reared at 18 ± 0.1 °C in current seawater conditions from 1 to 3 days post fertilisation before larvae (23 ± 0.78 per trial: average ±95% confidence interval) and each set of potential parents were genotyped on the basis of microsatellite loci^50^, and larval paternity assigned using Cervus v. 3.0.7^51^ at a greater than 95% level of confidence (n = 1273) [see also Supplementary Methods and Supplementary Table S7]. Trials involving some combinations of pairs and females had to be discarded if microsatellite genotyping could not clearly assign paternity (due to the presence of shared alleles or possible null alleles). This meant that the resulting larvae of trials with the eggs of either two or three females were genotyped for each pair (see Supplementary Table S8 for raw data).

### Statistical modelling

The faster male of a pair (based upon average sperm swimming speed (VCL) in current conditions) was selected as the ‘focal’ male and his competitor as the ‘rival’ male. Relative ejaculate traits were calculated as the focal male minus the rival male. We explored the influence of relative male ejaculate traits and seawater conditions on competitive fertilisation success in our paired trials using a generalized linear-mixed effects modelling (GLMM) approach. GLMM fixed effects were scaled around their mean value and the binomial error family and probit link were selected for the model structure. We accounted for the random effect of pair identity and included a second random term: a dispersion parameter, to account for overdispersion. Models were built using the following basic fixed effects structure:


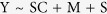


where Y = the proportion of larvae sired by the focal male, SC = seawater conditions, M = the relative proportion of motile sperm and S = relative average sperm swimming speed (VCL).

Once we had constructed a model containing main effects only, we built a series of models containing combinations of main effects and two-way interactions and finally a model containing all three two-way interactions between fixed effects. This resulted in the following GLMM fixed effects structures:


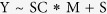



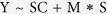



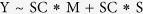



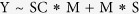



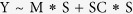



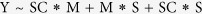


We repeated the construction of GLMMs containing different fixed effects structures where we substituted relative average sperm swimming speed (VCL) for three alternative relative speed terms to investigate whether faster sub-populations of sperm within a male’s ejaculate were associated with competitive fertilisation success. These included the average sperm swimming speed (VCL) of the fastest 1, 5 and 10% of motile sperm (see Supplementary Methods). This resulted in a total of 32 GLMMs.

We compared the performance of each constructed GLMM against our model selection criteria to identify the ‘best fitting’ model most supported by the paternity share data (see Supplementary Tables S2–S5 for outputs). Our primary selection criterion was a minimised Akaike information criterion corrected for small sample sizes (AICc)^52^. A maximised rounded Akaike weight [w_i_(AICc)] was the secondary selection criterion, which we directly interpreted as a conditional probability for each model. We only considered models with a ∆_i_(AICc) of less than 2 i.e. the difference between the AICc value of the i^th^ model in the selection and the minimum AICc value. Models with AICc values within 2 of the ‘best fitting’ model were considered statistically equivalent. However, the model which performed maximally against our selection criteria was selected to generate the graphs in Fig. 2 and test the significance of seawater conditions and relative male ejaculate traits on competitive fertilisation success in our paired trials.

Statistical analyses were conducted in R version 3.0.2^53^. Modelling was undertaken in the ‘lme4’ R package using the ‘glmer’ command. Model dispersion was checked in R (see Supplementary Methods for R code). GLMM performance against our selection criteria was compared using the ‘MuMIn’ R package and the ‘model.sel’ command. The conditional and marginal coefficient of determination was calculated for the ‘best fitting’ GLMM using the ‘MuMIn’ R package and the ‘r.squaredGLMM’ command. The fitted values and 95% confidence intervals found in Fig. 2 were generated using the ‘best fitting’ GLMM, the ‘effects’ R package and the ‘effect’ command. This allowed the influence of one significant interaction or main effect on paternity shares to be plotted, whilst minimising the influence of other terms marginal to the effect in question. All graphs were produced in GraphPad Prism 6^54^.

## Additional Information

**How to cite this article**: Campbell, A. L. *et al*. Ocean acidification changes the male fitness landscape. *Sci. Rep.* **6**, 31250; doi: 10.1038/srep31250 (2016).

## Supplementary Material

Supplementary Information

## Figures and Tables

**Table 1:** Parameter estimates for the ‘best fitting’ GLMM of the influence of relative ejaculate traits (calculated as the focal male – his rival male) and seawater conditions upon the proportion of larvae sired by the focal male in paired competitive fertilisation trials [significant terms are highlighted in bold (*p* ≤ 0.05)].

Fixed effects	Estimate (±SE)	df	Z value	Pr(>Z)
Intercept	0.037 ± 0.110	7	0.335	0.738
**Relative sperm swimming speed (VCL)**	**0.368** **±** **0.094**	**7**	**3.897**	**0.000**
**Seawater conditions (OA)**	**0.502** **±** **0.159**	**7**	**3.159**	**0.002**
**Relative percentage of motile sperm**	**0.474** **±** **0.165**	**7**	**2.868**	**0.004**
Relative percentage of motile sperm * seawater conditions (OA)	**−0.496** **±** **0.194**	**7**	**−2.559**	**0.010**

The model controlled for the random effects of pair identity and a dispersion parameter.
