# Supplementary material for: Ocean acidification changes the male fitness landscape
Source: Sci Rep. 2016 Aug 17;6:31250. doi: 10.1038/srep31250 (PMC4987666; doi:10.1038/srep31250)
Supplement: Supplementary Information [file srep31250-s1.pdf]

Ocean acidification changes the male fitness landscape

Anna L. Campbell[1], Don R. Levitan[2], David J. Hosken[3], Ceri Lewis[1]*

[1] College of Life and Environmental Sciences,

University of Exeter,

Geoffrey Pope Building,

Stocker Road, Exeter,

EX4 4QD, UK

[2] Department of Biological Science,

Florida State University,

King Life Sciences Building,

319 Stadium Drive,

Tallahassee, FL,

32306-1100, USA

[3] College of Life and Environmental Sciences,

University of Exeter, Cornwall Campus

Treliever Road

Penryn, Cornwall

TR10 9FE, UK

* Corresponding author e-mail: c.n.lewis@exeter.ac.uk

Supplementary Information:

Supplementary Table S1. Statistical output of Levene's test for homogeneity of variance in male ejaculate traits across seawater conditions. Significant terms are highlighted in bold ($p \leq 0.05$).

Ejaculate trait	df	F	Pr(>F)
**Percentage of motile sperm**	**41**	**13.264**	**0.001**
Average sperm swimming speed (VCL) of all motile sperm	41	0.005	0.945
Average straightness of sperm path (STR)	41	1.411	0.242
Average linearity of sperm path (LIN)	41	1.624	0.210

Supplementary Table S2. Performance of GLMMs containing the average sperm swimming speed (VCL) of all motile sperm as the relative speed term against our selection criteria. Models are ordered by ascending AICc values (a minimised AICc value was the primary selection criterion) and model dispersion is reported. The model with the highest performance against our selection criteria is highlighted in bold. [SW= seawater conditions, M= the relative proportion of motile sperm and S= relative average sperm swimming speed (VCL) of all motile sperm]

Model fixed effects	df	AICc	Δ_i(AICc)	w_i(AICc)	Dispersion
**SW * M + S**	**7**	**167.4**	**0.00**	**0.273**	**0.541**
SW + M + S	6	168.5	1.16	0.153	0.360
SW * M + M * S	8	172.2	4.79	0.025	0.603
SW * M + SW * S	8	172.3	4.94	0.023	0.583
SW + M * S	7	172.4	5.04	0.022	0.392
SW * S + M	7	172.7	5.36	0.019	0.391
SW * S + M * S	8	177.4	10.07	0.002	0.422
SW * M + M * S + SW * S	9	177.9	10.51	0.001	0.653

Supplementary Table S3. Performance of GLMMs containing the average sperm swimming speed (VCL) of the fastest 10 % of motile sperm as the relative speed term against our selection criteria. Models are ordered by ascending AICc values (a minimised AICc value was the primary selection criterion) and dispersion is reported. [SC= seawater conditions, M= the relative proportion of motile sperm and S= relative average sperm swimming speed (VCL) of the fastest 10 % of motile sperm]

Model fixed effects	df	AICc	Δ_i(AICc)	w_i(AICc)	Dispersion
SC + M + S	6	168.9	1.56	0.125	0.316
SC * M + S	7	169.6	2.22	0.090	0.429
SC + M * S	7	173.0	5.67	0.016	0.337
SC * S + M	7	173.3	5.95	0.014	0.337
SC * M + SC * S	8	174.6	7.22	0.010	0.457
SC * M + M * S	8	174.7	7.29	0.007	0.459
SC * C + M * S	8	178.1	10.75	0.001	0.361
SC * M + M * S + SC * S	9	180.5	13.14	0.000	0.492

Supplementary Table S4. Performance of GLMMs containing the average sperm swimming speed (VCL) of the fastest 5 % of motile sperm as the relative speed term against our selection criteria. Models are ordered by ascending AICc values (a minimised AICc value was the primary selection criterion) and dispersion is reported. [SC= seawater conditions, M= the relative proportion of motile sperm and S= relative average sperm swimming speed (VCL) of the fastest 5 % of motile sperm]

Model fixed effects	df	AICc	Δ_i(AICc)	w_i(AICc)	Dispersion
SC + M + S	6	169.6	2.19	0.092	0.304
SC * M + S	7	170.9	3.56	0.046	0.390
SC * S + M	7	173.8	6.46	0.011	0.329
SC + M * S	7	173.9	6.49	0.011	0.322
SC * M + M * S	8	176.0	8.60	0.004	0.422
SC * M + A * S	8	176.0	8.60	0.004	0.421
SC * S + M * S	8	178.8	11.47	0.001	0.349
SC * M + M * S + SC * S	9	181.8	14.46	0.000	0.462

Supplementary Table S5. Performance of GLMMs containing the average sperm swimming speed (VCL) of the fastest 1 % of motile sperm as the relative speed term against our selection criteria. Models are ordered by ascending AICc values (a minimised AICc value was the primary selection criterion) and dispersion is reported. [SC= seawater conditions, M= the relative proportion of motile sperm and S= relative average sperm swimming speed (VCL) of the fastest 1 % of motile sperm]

Model fixed effects	df	AICc	Δ_i(AICc)	w_i(AICc)	Dispersion
SC + M + S	6	171.9	4.49	0.029	0.274
SC * M + S	7	173.9	6.57	0.010	0.330
SC * S + M	7	174.7	7.37	0.007	0.321
SC + M * S	7	176.3	8.89	0.003	0.292
SC * M + SC * S	8	178.5	11.10	0.001	0.369
SC * M + M * S	8	178.9	11.56	0.001	0.358
SC * S + M * S	8	179.4	12.02	0.001	0.363
SC * M + M * S + SC * S	9	183.8	16.44	0.000	0.423

Supplementary Table S6. Measured and calculated (CO2SYS[1]) seawater parameters for experimental seawater conditions [experiments took place on 12.06.13 (a) and 12.06.14 (b)].

**Conditions**	**Measured parameters**				**Calculated parameters**					
	T (°C)	S	**pH**	**DIC (µmol kg^{-1})**	**TA (µmol kg^{1})**	**$p$$CO_2$ (µatm)**	**HCO_3^- (µmol kg^{-1})**	**CO_3^{2-} (µmol kg^{-1})**	**Ω Ca**	**Ω Ag**
**Current[a]**	14 ± 0.1	35	8.19	2513.0	2770.8	446.5	2299.0	196.8	4.69	3.01
**Current[b]**	14 ± 0.1	34.9	8.16	2512.7	2754.9	476.0	2308.3	186.1	4.43	2.84
**OA[a]**	14 ± 0.1	35	7.73	2638.0	2688.9	1407.0	2509.4	74.4	1.77	1.14
**OA[b]**	14 ± 0.1	34.9	7.69	2593.0	2628.3	1528.7	2467.9	66.2	1.58	1.01

Supplementary Table S7. Details of microsatellite markers used in the study (n=22 adult sea urchins).

Marker	Size range of alleles in base pairs (study observations combined with published data[2])	Estimated number of alleles identified in study population
PHIST	338-473	26
PIB	82-438	11
PIC	331-421	16
PIL	187-277	15
PI15	96-190	22
PIT	160-272	13

Supplementary Table S8. Raw data table of the percentage of larvae sired by the focal male in paired competitive fertilisation trials and relative ejaculate traits in current and simulated OA conditions.

Seawater conditions	Pair ID	Relative average sperm swimming speed (VCL: μms^{-1})	Relative percentage sperm motility (%)	Female ID	Percentage of genotyped larvae sired by the focal male (%)
Current	1	70.00	6.42	2	73.08
				4	89.29
	2	40.95	16.07	1	50.00
				3	68.00
				4	69.23
	3	62.75	3.69	1	64.29
				2	42.86
				4	81.82
	4	56.94	13.15	2	52.38
				3	66.67
				4	69.57
	5	55.61	9.63	1	62.50
				2	57.14
				4	68.00
	6	17.72	0.46	1	36.00
				3	39.13
				4	44.83
	7	20.1	3.45	2	20.00
				3	37.50
				4	60.00
	8	1.66	26.34	5	72.22
				6	52.63
	9	43.14	6.26	5	55.00
				6	68.18
	10	48.95	23.83	5	86.36
				6	85.00
	11	52.73	-5.20	5	90.48
				6	91.30
OA	1	-52.09	-17.35	2	47.83
				3	26.92
	2	36.00	9.18	1	95.24
				3	78.95
				4	100.00
	3	34.77	-1.76	1	69.57
				2	79.17
				4	79.17
	4	-10.09	-22.13	2	57.14
				3	46.43
				4	71.43
	5	45.62	26.76	1	47.83
				2	52.94
				4	61.11
	6	-35.94	-2.15	1	34.62
				3	73.08
				4	21.74
	7	38.88	1.88	2	69.57
				3	79.17
				4	50.00
	8	23.32	10.87	5	61.90
				6	27.27
	9	-4.92	18.93	5	47.62
				6	38.89
	10	14.99	33.69	5	85.00
				6	94.44
	11	35.5	13.80	5	76.19
				6	91.30

Supplementary Methods:

Seawater pCO$_2$ manipulation

Artificial seawater (Aquamarine Ltd) was filtered to 1 µm and made up to a salinity of 35 salinity units (psu). Additional salinity measurements were made using a Mettler Toledo SG7 SevenGo pro conductivity meter to an accuracy of ± 0.1 psu. Seawater pCO$_2$ was manipulated using a computerised control system (NBS scale, AquaMedic, Germany) which regulated pH via a solenoid valve and CO$_2$ injector in conjunction with vigorous aeration. Additional seawater pH measurements were taken using a Metrohm (827 pH lab) pH$_{NBS}$ electrode and NBS buffers and once at the desired pH (± 0.02) seawater was stored in sealed containers lacking an airspace for use within 1 hour of collection. A seawater pH value of 7.70 was targeted in the OA treatment to represent near-future OA as projected according to scenario RCP 8.5 IPCC WGI AR5[3,4]. The artificial seawater used in this study had a relatively high alkalinity compared to natural seawater, and this additional buffering capacity resulting in a slightly higher pCO$_2$ (1468 µatm) in order to reach the Intergovernmental Panel on Climate Change (IPCC) relevant pH value than would be required for open water natural seawater. However, this level of pCO$_2$ is relevant for coastal values[unpublished data,5]. Dissolved inorganic carbon (DIC) analysis was carried out upon seawater samples collected during each experiment using a custom built system described by Friederich, et al. [6] and following the methodology found in Lewis, et al. [7]. This system allowed the measurement of seawater DIC with a precision of ± 3 µM. Additional seawater parameters; total alkalinity and pCO$_2$, were calculated using CO2SYS[1] according to Findlay, et al. [8] and using the pH, salinity and DIC measurements along with the NBS scale and Dickson standards.

Assessment of ejaculate characteristics

Following incubation a small sub-sample of diluted sperm was transferred to Leja 20 mm standard counting chambers for analysis. Motility assessment took place using a Microptic Sperm Class Analyser (SCA®: Microm, UK) fitted with a Nikon Eclipse 50i negative phase contrast microscope (100 x magnification) and a Peltier cooled stage which was operated at 14 ± 0.1 °C. Images were captured at a rate of 100 frames s^{-1} with individual sperm tracked for 0.5 seconds. A minimum of 500 sperm were tracked in each sample and a range of CASA derived motility parameters calculated for each sperm tracked using the SCA® Motility and concentration module. Samples were analysed once (one technical replicate) but we analysed a large number of sperm per sample (> 500) as previous work has indicated a high within-sample repeatability for CASA parameters using this approach[9]. Threshold values of > 10 µm s^{-1} curvilinear velocity (VCL) and > 3.2 µm s^{-1} straight line velocity (VSL) were used to remove the influence of immotile sperm moving via capillary drift from subsequent analysis and to determine the percentage of motile sperm within each sample (representative of the ejaculate). We also calculated several additional speed parameters for each male; the average VCL of the fastest 1, 5 and 10 % of sperm. To achieve this sperm were ordered by ascending speed and split into speed percentiles. Sperm path linearity (LIN) and sperm path straightness (STR) were calculated by the SCA® CASA System [LIN: VSL/VCL and STR: VSL/ average sperm path velocity (VAP)].

Competitive fertilisations and larval paternity assignment

Fertilisations were allowed to proceed for 30 minutes in 20 ml of treatment seawater before fertilisation beakers were topped up to 350 ml with current seawater and incubated overnight at 14 ± 0.1 °C. Beakers were gently aerated from twelve hours post fertilisation and the incubation

temperature increased to 18 ± 0.1 °C to enhance larval development. Larvae were fed an appropriate ration of *Isochrysis* algal paste on days 2 and 3. Larval development was terminated at the end of day 3 when cultures were filtered through a 60 µm mesh and larvae were re-suspended in 95 % ethanol and stored in microcentrifuge tubes at -20 °C alongside a 1 cm^2 section of gonad tissue dissected from each potential parent. Detailed methodology on paternity assignment can be found in Levitan [10]. In short DNA was extracted from individual larvae or adult gonad tissue, diluted and then amplified using fluorescently labelled primers via the polymerase chain reaction (PCR). Following amplification PCR products were genotyped using an Applied Biosciences 3730xl DNA Analyzer. The reaction products were visualised and scored for six potential microsatellite loci (see Table S4 for information on microsatellite markers) using GeneMapper v. 3.5 software (Applied Biosystems, CA, USA). In order to assign paternity through the identification of inherited markers, a minimum of 2 of the most diagnostic loci for each potential set of parents were selected and screened for in the larvae.

R code

To calculate dispersion of a GLMM object:

```
overdisp_fun <- function(model) {
  ## number of variance parameters in
  ##   an n-by-n variance-covariance matrix
  vpars <- function(m) {
    nrow(m)*(nrow(m)+1)/2
  }
  model.df <- sum(sapply(VarCorr(model),vpars))+length(fixef(model))
  rdf <- nrow(model.frame(model))-model.df
  rp <- residuals(model,type="pearson")
  Pearson.chisq <- sum(rp^2)
  prat <- Pearson.chisq/rdf
  pval <- pchisq(Pearson.chisq, df=rdf, lower.tail=FALSE)
  c(chisq=Pearson.chisq,ratio=prat,rdf=rdf,p=pval)
}
#command to calculate the dispersion of a GLMM object
overdisp_fun(GLMMobject)
```

Supplementary References:

1. Pierrot D, Lewis E, Wallace D. MS Excel program developed for CO_2 system calculations. *ORNL/CDIAC-105a Carbon Dioxide Information Analysis Center, Oak Ridge National Laboratory, US Department of Energy, Oak Ridge, Tennessee* 2006.

2. Calderón I, Turon X, Pascual M. Isolation of nine nuclear microsatellites in the common Mediterranean sea urchin, *Paracentrotus lividus* (Lamarck). *Molecular Ecology Resources* 2009, **9**(4)**:** 1145-1147.

3. Stocker T, Qin D, Plattner G, Tignor M, Allen S, Boschung J*, et al.* IPCC, 2013: climate change 2013: the physical science basis. Contribution of working group I to the fifth assessment report of the intergovernmental panel on climate change. 2013.

4. Meinshausen M, Smith SJ, Calvin K, Daniel JS, Kainuma M, Lamarque J*, et al.* The RCP greenhouse gas concentrations and their extensions from 1765 to 2300. *Climatic Change* 2011, **109**(1-2)**:** 213-241.

5. Melzner F, Thomsen J, Koeve W, Oschlies A, Gutowska MA, Bange HW*, et al.* Future ocean acidification will be amplified by hypoxia in coastal habitats. *Marine Biology* 2013, **160**(8)**:** 1875-1888.

6. Friederich G, Walz P, Burczynski M, Chavez F. Inorganic carbon in the central California upwelling system during the 1997–1999 El Niño–La Niña event. *Progress in Oceanography* 2002, **54**(1)**:** 185-203.

7. Lewis C, Clemow K, Holt WV. Metal contamination increases the sensitivity of larvae but not gametes to ocean acidification in the polychaete *Pomatoceros lamarckii* (Quatrefages). *Marine Biology* 2012, **160**(8)**:** 2089-2101.

8. Findlay HS, Artioli Y, Moreno Navas J, Hennige SJ, Wicks LC, Huvenne VA*, et al.* Tidal downwelling and implications for the carbon biogeochemistry of cold-water corals in relation to future ocean acidification and warming. *Global Change Biology* 2013, **19**(9)**:** 2708-2719.

9. Fitzpatrick JL, Simmons LW, Evans JP. Complex patterns of multivariate selection on the ejaculate of a broadcast spawning marine invertebrate *Evolution* 2012, **66**(8)**:** 2451-2460.

10. Levitan DR. Gamete traits influence the variance in reproductive success, the intensity of sexual selection, and the outcome of sexual conflict among congeneric sea urchins. *Evolution* 2008, **62**(6)**:** 1305-1316.

11. R Core Team. R: A language and environment for statistical computing. R Foundation for Statistical Computing: Vienna, Austria, 2013.
